# The effect of antipsychotics on glutamate levels in the anterior cingulate cortex and clinical response: A ^1^H-MRS study in first-episode psychosis patients

**DOI:** 10.3389/fpsyt.2022.967941

**Published:** 2022-08-11

**Authors:** Uzma Zahid, Robert A. McCutcheon, Faith Borgan, Sameer Jauhar, Fiona Pepper, Matthew M. Nour, Maria Rogdaki, Martin Osugo, Graham K. Murray, Pamela Hathway, Robin M. Murray, Alice Egerton, Oliver D. Howes

**Affiliations:** ^1^Department of Psychosis Studies, Institute of Psychiatry, Psychology and Neuroscience, King’s College London, London, United Kingdom; ^2^Department of Neuroimaging, Institute of Psychiatry, Psychology and Neuroscience, King’s College London, London, United Kingdom; ^3^Department of Clinical and Movement Neurosciences, Queen Square Institute of Neurology, University College London Centre, London, United Kingdom; ^4^Max Planck University College London Centre for Computational Psychiatry and Ageing Research, London, United Kingdom; ^5^Wellcome Trust Centre for Neuroimaging, University College London, London, United Kingdom; ^6^Department of Child and Adolescent Psychiatry, Institute of Psychiatry, Psychology and Neuroscience, King’s College London, London, United Kingdom; ^7^Department of Psychiatry, University of Cambridge, Cambridge, United Kingdom; ^8^Institute of Clinical Sciences, Faculty of Medicine, Imperial College London, London, United Kingdom; ^9^H. Lundbeck UK, Valby, Denmark

**Keywords:** spectroscopy, NMDA, imaging and schizophrenia, CSF-correction, longitudinal, glutamate

## Abstract

**Introduction:**

Glutamatergic dysfunction is implicated in the pathophysiology of schizophrenia. It is unclear whether glutamatergic dysfunction predicts response to treatment or if antipsychotic treatment influences glutamate levels. We investigated the effect of antipsychotic treatment on glutamatergic levels in the anterior cingulate cortex (ACC), and whether there is a relationship between baseline glutamatergic levels and clinical response after antipsychotic treatment in people with first episode psychosis (FEP).

**Materials and methods:**

The sample comprised 25 FEP patients; 22 completed magnetic resonance spectroscopy scans at both timepoints. Symptoms were assessed using the Positive and Negative Syndrome Scale (PANSS).

**Results:**

There was no significant change in glutamate [baseline 13.23 ± 2.33; follow-up 13.89 ± 1.74; t(21) = −1.158, *p* = 0.260], or Glx levels [baseline 19.64 ± 3.26; follow-up 19.66 ± 2.65; t(21) = −0.034, *p* = 0.973]. There was no significant association between glutamate or Glx levels at baseline and the change in PANSS positive (Glu *r* = 0.061, *p* = 0.777, Glx *r* = −0.152, *p* = 0.477), negative (Glu *r* = 0.144, *p* = 0.502, Glx *r* = 0.052, *p* = 0.811), general (Glu *r* = 0.110, *p* = 0.607, Glx *r* = −0.212, *p* = 0.320), or total scores (Glu *r* = 0.078, *p* = 0.719 Glx *r* = −0.155, *p* = 0.470).

**Conclusion:**

These findings indicate that treatment response is unlikely to be associated with baseline glutamatergic metabolites prior to antipsychotic treatment, and there is no major effect of antipsychotic treatment on glutamatergic metabolites in the ACC.

## Introduction

Psychotic illnesses such as schizophrenia are characterised by positive symptoms such as delusions and hallucinations, negative symptoms such as anhedonia and blunted affect, and cognitive deficits (1). The disruption of dopaminergic signalling has been identified as a core component of the neurobiology of psychosis (2, 3). In support of this, previous studies have shown an association between antipsychotic striatal D_2_ occupancy and clinical response (4).

Glutamatergic dysfunction has also been implicated in the pathophysiology of schizophrenia (5). Glutamate is an excitatory neurotransmitter, with two prominent classes of receptors: ionotropic and metabotropic. There is a growing body of evidence suggesting that hypofunction of the ionotropic glutamate receptor *N*-methyl-D-aspartate (NMDA) plays a role in the pathophysiology of schizophrenia (5, 6). For example, the NMDA receptor antagonist ketamine has been shown to induce negative symptoms and cognitive deficits, paralleling deficits seen in schizophrenia (7, 8). NMDA antagonists may reduce GABAergic interneuron functioning, leading to an increased release of neurotransmitters such as dopamine and glutamate (9–11). Thus, striatal dopaminergic hyperactivity in schizophrenia may be secondary to alterations in the glutamatergic system (12).

Proton magnetic resonance spectroscopy (^1^H-MRS) enables the *in vivo* quantification of brain glutamate levels (13). Using this technique, ketamine has been shown to increase glutamate measures in the anterior cingulate cortex (ACC) in healthy volunteers (14). Findings from cross-sectional ^1^H-MRS studies in patients with schizophrenia have shown that glutamate levels vary depending on whether patients demonstrate a clinical response to antipsychotic treatment. Demjaha et al. found that glutamate levels in the ACC were elevated in the treatment resistant (*n* = 6) but not treatment responsive patients (*n* = 8) with non-affective psychosis (15). Similarly, Mouchlianitis et al. (16) compared patients with non-affective psychosis that were either treatment responsive or treatment resistant. They found increased glutamate levels in the ACC of treatment resistant patients (*n* = 21) relative to treatment responsive patients (*n* = 20) (16). Egerton et al. found that ACC glutamate levels were elevated in patients with non-affective psychosis who were treatment resistant (*n* = 44) relative to those who were treatment responsive (*n* = 48) (17). However, Goldstein et al. Showed no group differences in ACC glutamate or Glx (combined signal of glutamate and glutamine) levels when comparing non-affective psychosis patients who were treatment responsive (*n* = 15), clozapine-responsive (*n* = 16), and clozapine-resistant (*n* = 11) (18). More recently, Tarumi et al. (19) showed no group difference in dorsal ACC (dACC) and caudate Glx levels between patients with non-affective psychosis who were either severely treatment resistant (*n* = 28) or treatment responsive (*n* = 31). Interestingly dACC Glx levels were higher in the treatment resistant group than in the healthy volunteer group (*n* = 29) (18). But, as these studies were cross-sectional in design, the outcome and exposure variables were measured at the same time, making it difficult to establish causal relationships. Cross-sectional studies also make it difficult to determine the stability of response and resistance status. Finally, these studies have included patients who have had prolonged antipsychotic exposure, which might have influenced brain glutamate levels (20).

To address these issues, several longitudinal ^1^H-MRS studies have investigated the effect of antipsychotic treatment on glutamate and Glx levels in schizophrenia (21, 22). De la Fuente-Sandoval et al. found reduced glutamate levels in the striatum of antipsychotic naïve patients during their first non-affective psychosis episode after 4 weeks of antipsychotic treatment (*n* = 24) (23). Egerton et al. reported a reduction in ACC glutamate levels of minimally treated patients during their first non-affective psychosis episode after 4 weeks of antipsychotic treatment (*n* = 46) (24). Conversely, Kraguljac et al. reported no change in ACC or hippocampal glutamate levels in unmedicated non-affective psychosis patients (*n* = 61), after 6 weeks of antipsychotic treatment (25). A limitation of the majority these longitudinal studies, which could explain the heterogeneity in results, is that they report glutamate scaled to creatine (Cr) (25–30). The Cr peak is often used as a concentration reference in human ^1^H-MRS studies, where metabolites are reported as ratios to Cr (31). Recently, however, Merritt et al., in a mega-analysis of schizophrenia studies, reported a trend toward lower Cr levels in patients with schizophrenia in the medial frontal cortex, including the ACC, suggesting that the use of Cr as a reference in schizophrenia research could yield inaccurate findings and that scaling to water and correcting for cerebrospinal fluid (CSF) are preferable to avoid this bias (20).

Considering these methodological limitations, we aimed to investigate in a FEP sample whether there is a relationship between baseline glutamate and Glx levels scaled to water and corrected for CSF in the ACC and clinical response at follow-up after antipsychotic treatment. Our secondary aim was to investigate whether antipsychotics alter brain glutamate and Glx levels scaled to water and corrected for CSF, in the ACC at follow-up. We hypothesised that (1) glutamate and Glx levels at baseline would be directly associated with treatment response following antipsychotic medication; (2) glutamate and Glx levels will decrease after antipsychotic administration relative to baseline.

## Materials and methods

This study was approved by the East of England-Cambridge East NHS Research Ethics Committee. All participants provided informed written consent prior to participation. The baseline ^1^H-MRS data have been reported previously (13, 32).

### Participants

Patients were recruited from early intervention psychosis services in London. Inclusion criteria were a diagnosis of schizophrenia or other psychotic disorders according to ICD-10 criteria (33), fulfilling criteria for having a first episode of psychosis [first treatment contact (34)] requiring treatment with antipsychotic medication, and being antipsychotic naive, antipsychotic free or minimally treated (taking antipsychotic medication for 2 weeks or less). Whilst other studies have used 3 weeks wash-out or oral antipsychotics (35) we defined subjects as being antipsychotic free if they had not taken any antipsychotic medication for at least 6 weeks (oral) or 6 months (depot, if relevant) to be conservative. Details of their prior antipsychotic medication and antipsychotic treatment between baseline and follow-up is available in Table 1. Chlorpromazine-equivalent doses were calculated for prior antipsychotic exposure using a previously described method (36). For lurasidone and amisulpride, we calculated the chlorpromazine-equivalent dose using the method described by Leucht et al. (37) and using data from the Maudsley Prescribing Guidelines, because these are not covered by Andreasen et al. (36). Exclusion criteria for all subjects were history of significant head trauma, dependence on illicit substances or alcohol, medical comorbidity (other than minor illnesses), current use of mood stabilisers–owing to effects on glutamate, and contraindications to MRI scanning. Ethnicity was self-reported, and level of education information collected using a sociodemographic schedule.

**TABLE 1:** Socio-demographic and clinical characteristics of participants.

		Patients (*n* = 25)
**Sex**		
	Male	19
	Female	6
Years in Education, years (Mean [SD])	13.62 (3.33)	
**Ethnicity**		
	White	11
	Black	8
	Asian	2
	Mixed/Other	4
**Medication Status at Baseline**		
	Antipsychotic naive	12
	Minimally treated	6
	*Amisulpride*	2
	*Risperidone*	1
	*Missing*	3
	**Antipsychotic free**	7
	*Risperidone*	1
	*Risperidone and pipothiazine*	1
	*Risperidone and paliperidone*	1
	*Aripiprazole*	1
	*Amisulpride and olanzapine*	1
	*Missing*	2
**Medication Between Baseline and Follow-Up**		
**Antipsychotic naïve**		
	*Amisulpride*	3
	*Aripiprazole*	2
	*Amisulpride and quetiapine*	1
	*Paliperidone, quetiapine, and olanzapine*	1
	*Risperidone*	1
	*Quetiapine*	1
	*Olanzapine*	1
	**Minimally treated**	
	*Amisulpride*	2
	*Aripiprazole*	1
	*Aripiprazole and risperidone*	1
	*Missing*	1
	**Antipsychotic free**	
	*Risperidone*	3
	*Amisulpride*	1
	*Aripiprazole and olanzapine*	1
	*Lurasidone*	1
	*Missing*	1
Duration of Untreated Psychosis, months (Mean [SD])	18.63 (17.55)*	
**PANSS Baseline (Mean [SD])**		
	Positive	19.29 (5.88)
	Negative	16.17 (6.46)
	General	36.25 (10.66)
	Total	71.71 (19.71)
PANSS Follow-Up (Mean [SD])		
	Positive	13.00 (5.28)
	Negative	12.75 (5.97)
	General	27.00 (8.82)
	Total	52.75 (18.05)

*Mean duration of psychosis calculated from available data (n = 19).

### Clinical assessment

All patients were clinically assessed at baseline and reassessed after being compliant with antipsychotic treatment at a therapeutic dose as specified in the Maudsley Prescribing Guidelines (38) for a minimum of 4 weeks, before determining treatment response. Four weeks was chosen as the minimum duration of treatment based on evidence that most therapeutic responses to antipsychotic medication occur within 4 weeks (39, 40) including in first-episode psychosis (41). Moreover, non-response before 4 weeks is a predictor of subsequent non-response (40).

The choice of antipsychotic commenced was determined by the treating clinician in discussion with the patient as per standard clinical practice. Prior use of other psychotropic medication (e.g., antidepressants and benzodiazepines) was not an exclusion criterion for the study; however, current use of psychotropic medication (antidepressant or mood stabilizer medication) during the study period was an exclusion criterion. To assess concordance with antipsychotic medication, we used a multisource approach, requiring evidence of adequate adherence on at least two of the following: antipsychotic plasma levels, pharmacy, and electronic medical dispensing records, or reports from the patient and an independent source (family member/caregiver or health care professional) (42). Adequate concordance was defined as taking a minimum of 80% of prescribed doses, in line with consensus recommendations (43).

Symptoms were rated at baseline and follow-up using the Positive and Negative Syndrome Scale (PANSS) (44). The duration of illness was calculated from the onset of the first psychotic symptoms to the initiation of antipsychotic treatment as previously described (45).

### Magnetic resonance spectroscopy (^1^H-MRS)

#### ^1^H-MRS acquisition

All scans were acquired on a General Electric (Milwaukee, Wisconsin) Signa HDxt 3Tesla MRI scanner using an 8-coil head channel, as described previously (13). For the voxel placements, 3D coronal inversion recovery prepared spoiled gradient echo (IR-SPGR) scans were acquired, followed by auto pre-scans for optimisation of water suppression and shimming. ^1^H-MRS spectra were acquired for the anterior cingulate (20 × 20 × 20 mm^3^). The placement of the anterior cingulate voxel was based on the midline sagittal localizer with the centre of the 20 mm × 20 mm × 20 mm voxel placed 13 mm above the anterior portion of the genu of the corpus callosum, perpendicular to the anterior commissure-posterior commissure line to minimize the inclusion of white matter and cerebral spinal fluid (CSF) (see Supplementary Figure 1 for sample voxel placement). Finally, the ^1^H-MRS spectra [Point RESolves Spectroscopy (PRESS), TE = 30 ms, TR = 2 s] were obtained through the PROton Brain Examination (PROBE) sequence by GE, which includes water suppression. The spectra were an average of 96 water suppressed acquisitions. Sixteen transients were also acquired without water suppression for use with water-referencing and eddy-current correction.

#### ^1^H-MRS quantification

Raw metabolite concentrations were estimated using LCModel version 6.3-0L,^1^ which estimates the concentrations of 16 metabolites (L-alanine, aspartate, creatine, phosphocreatine, GABA, glucose, Glutamine, glutamate, glycerophosphocholine, glycine, myo-inositol, L-lactate, N-acetylaspartate, N-acetylaspartylglutamate, phosphocholine, and taurine) by fitting the output to a standard basis set acquired experimentally. As described previously (13), metabolite analyses were restricted to spectra with linewidth (full-width at half-maximum; FWHM) ≤ 0.1 ppm, Cramér-Rao lower bounds (CRLB) for glutamate ≤ 20%, signal to noise ratio ≥ 5. The data are not truncated. In-house scripts written in Python were used to identify the relative distribution of white matter, grey matter, and cerebrospinal fluid in the 8 cm^3^ voxel prescribed to the anterior cingulate cortex. The following correction was subsequently applied to correct for cerebrospinal fluid within the 8 cm^3^ voxel, where M = raw metabolite value, WM = white matter fraction and GM = grey matter fraction and CSF = cerebrospinal fluid fraction (46).


$$M\,c\,o\,r\,r=M\,\left(\frac{W\,M+\left(G\,M\times 1.21\right)+\left(C\,S\,F\times 1.55\right)}{\left(W\,M+G\,M\right)}\right)$$

In the equation, the numerator accounts for the fraction of each tissue type within the voxel, corrected by the water concentration in the tissue type. The denominator corrects for the assumption that CSF does not contain metabolites. No correction was applied for relaxation times, except for assuming the tissue water T2 = 80 ms. We report metabolite values scaled to water, as opposed to creatine, based on previous literature indicating that creatine levels are lower in patients with schizophrenia relative to healthy volunteers (47).

### Statistical analysis

Statistical analyses were performed using SPSS, version 25, and significance set to *p* < 0.05 (two-tailed). Normality of distribution was assessed using Shapiro–Wilk test. To test the hypothesis glutamate and Glx levels at baseline would be associated with treatment response following antipsychotic medication, Pearson’s correlation coefficient was calculated for glutamate and Glx levels at baseline and the percentage change in the PANSS score at follow-up. We carried out exploratory analyses investigating the association between the change in glutamate and Glx levels and the change in the PANSS score. Additionally, as cross-sectional studies report the association between endpoint glutamate and Glx levels and the PANSS score, we make available the results for this association in the present study. Pearson’s correlation coefficient was calculated for both exploratory analyses. To test the hypothesis that glutamate and Glx levels will decrease after antipsychotic administration relative to baseline we conducted a paired samples *t*-test. Quantitative variables are presented as mean ± standard deviation (SD). Additionally, we carried out an exploratory analysis in participants who were antipsychotic-naïve and antipsychotic free, excluding minimally treated participants, to see whether antipsychotic treatment was associated with longitudinal change in glutamatergic measures. Finally, Bayesian statistical analyses were conducted using JASP (JASP Team, 2021) to help quantify the relative evidence of the null and alternative hypotheses and support inferences (48, 49). We used JASP default priors: for a paired *t*-test, the prior was determined by a Cauchy distribution centred on a zero-effect size and a width/scale of 0.707; for correlation, the prior was that any correlation between −1 and 1 was equally likely. Bayes Factor (BF_10_) and corresponding credible intervals are provided.

Percentage changes for PANSS were calculated adjusting for minimum scores (7 for positive and negative symptom sub-scales, 30 for total symptoms) as shown here for the PANSS positive symptom subscale:


$$\;\;\text{\% change in positive PANSS}=\;\,\frac{\left(\left(\mathrm{baseline}\,\mathrm{score}-7\right)-\left(\mathrm{follow}-\mathrm{up}\,\mathrm{score}-7\right)\right)}{\left(\mathrm{baseline}\,\mathrm{score}-7\right)}\times 100$$

## Results

### Demographics

Demographic details of participants are given in Table 1. The sample comprised 25 first episode psychosis patients, 12 antipsychotic-naïve, 6 minimally treated, and 7 antipsychotic-free individuals. ICD-10 diagnoses at baseline were schizophrenia (*n* = 15) and bipolar disorder (*n* = 10). We acquired follow-up ^1^H-MRS scans from 22 participants in our sample, these are included in the baseline vs. follow-up analysis. We make available the FWHM, CRLB, SNR, and tissue fractions for both baseline and follow-up in the Supplementary Table 1.

### Glutamate and Glx levels before and after antipsychotic administration

**FIGURE 1:**
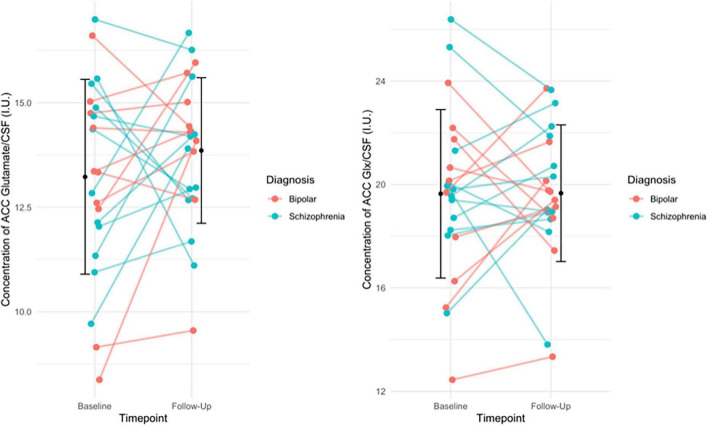
**(Left)** The individual change in glutamate levels from baseline to follow-up, with group mean (SD) of glutamate levels at baseline and follow-up (black circle and error bars). Individual change in the figure is stratified by diagnosis. Results of the paired *t*-test indicated no significant difference in glutamate levels over time (*p* = 0.260). **(Right)** The individual change in Glx levels from baseline to follow-up, with the group mean (SD) Glx levels at baseline and follow-up (black circles and error bars). Individual change in the figure is stratified by diagnosis. Results of the paired *t*-test indicated no significant difference in Glx levels over time (*p* = 0.973).

There was no significant change between baseline (13.23 ± 2.33) and follow-up glutamate levels ([13.89 ± 1.74]; t(21) = −1.158, *p* = 0.260) or between baseline (19.64 ± 3.26) and follow-up Glx levels ([19.66 ± 2.65]; t(21) = −0.034, *p* = 0.973, see Figure 1). Additionally, there was no significant change between baseline and follow-up glutamate and Glx levels when we excluded people who had been minimally treated. The results from this exploratory analysis are available in the Supplementary material. To quantify our null findings, we conducted Bayesian repeated measures *t*-tests. The resulting BF_10_ for baseline and follow up glutamate levels was 0.403 (95% CI: 0.623–0.178), indicating anecdotal evidence in favour of the null hypothesis of no change over time. The resulting BF_10_ for baseline and follow up Glx levels was 0.223 (95% CI: 0.397−0.384), indicating moderate evidence in favour of the null hypothesis of no change over time.

### Association between baseline glutamate and Glx levels and the change in PANSS sub-scales scores

**FIGURE 2:**
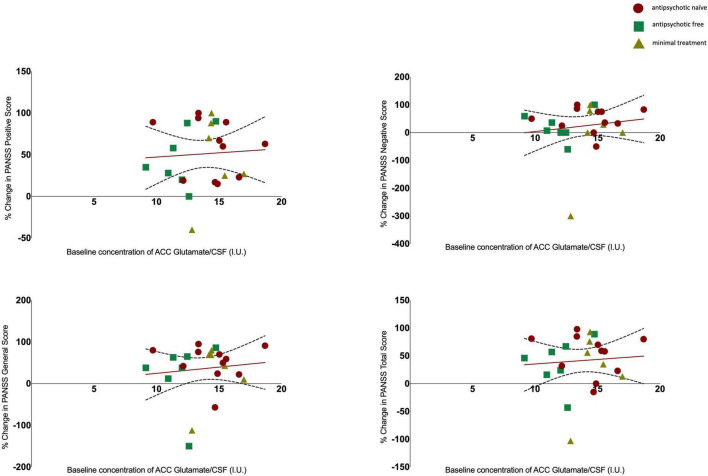
Relationship between glutamate levels and the percentage change in PANSS positive (*r* = 0.061, *p* = 0.777), negative (*r* = 0.144, *p* = 0.502), general (*r* = 0.110, *p* = 0.607), and total scores (*r* = 0.078, *p* = 0.719), with 95% confidence intervals derived from the line of best fit. Individuals are stratified by medication status, antipsychotic naïve (circle), antipsychotic free (square), minimal treatment (triangle).

**FIGURE 3:**
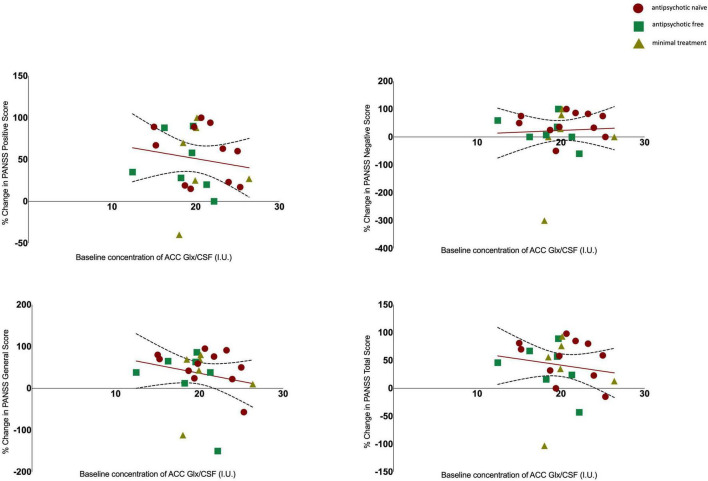
Relationship between Glx levels and the percentage change in PANSS positive (*r* = −0.152, *p* = 0.477), negative (*r* = 0.052, *p* = 0.811), general (*r* = −0.212, *p* = 0.320) and total scores (*r* = −0.155, *p* = 0.470) with 95% confidence intervals derived from the line of best fit. Individuals are stratified by medication status, antipsychotic naïve (circle), antipsychotic free (square), minimal treatment (triangle).

There was no significant association between glutamate levels at baseline and the change in PANSS positive scores (*r* = 0.061, *n* = 24, *p* = 0.777), the change in PANSS negative scores (*r* = 0.144, *n* = 24, *p* = 0.502) the change in PANSS general scores (*r* = 0.110, *n* = 24, *p* = 0.607) or the change in PANSS total scores (*r* = 0.078, *n* = 24, *p* = 0.719, see Figure 2). There was no significant association between Glx levels at baseline and the change in PANSS positive scores (*r* = −0.152, *n* = 24, *p* = 0.477), the change in PANSS negative scores (*r* = 0.052, *n* = 24, *p* = 0.811), the change in PANSS general scores (*r* = −0.212, *n* = 24, *p* = 0.320) or the change in PANSS total scores (*r* = −0.155, *n* = 24, *p* = 0.470, see Figure 3). As evident from the figures, an outlier value was present in the negative symptom scores; hence, we ran a sensitivity analysis excluding the observation containing the outlier. Findings show there was still no significant association between the change in PANSS negative scores and either glutamate levels (*r* = 0.127, *n* = 23, *p* = 0.563) or Glx levels (*r* = −0.101, *n* = 23, *p* = 0.647) at baseline. Furthermore, there was no significant association between the change in glutamate and Glx levels from baseline to follow-up and the change in PANSS scores from baseline to follow-up, and there was no significant association between glutamate levels at follow-up and the follow-up PANSS scores. The results from these exploratory analyses are available in the Supplementary material. To quantify our null findings, we conducted Bayesian correlations. The resulting BF_10_ for baseline glutamate levels and the change in PANSS positive (0.263), negative (0.313), general (0.287), and total (0.269) indicate moderate evidence in favour of the null hypothesis of no associations between glutamate levels and symptoms. The resulting BF_10_ for baseline Glx levels and the change in PANSS positive (0.322), negative (0.260), general (0.404), and total (0.324) indicate moderate to anecdotal evidence in favour of the null hypothesis of no association.

### Cr levels before and after antipsychotic administration

There was no significant change between baseline (6.25 ± 0.57) and follow-up creatine levels ([6.39 ± 0.55]; t(21) = 1.121, *p* = 0.275).

## Discussion

In a sample of FEP patients, we investigated whether there is a relationship between baseline ACC glutamate and Glx levels corrected for CSF and subsequent clinical response after antipsychotic treatment, and whether antipsychotics alter ACC glutamate and Glx levels corrected for CSF. No effect of antipsychotic treatment on glutamate and Glx levels in the ACC was found, and the therapeutic effects were not associated with glutamatergic levels measured before antipsychotic administration. Our findings are consistent with previous studies that have found no effect of antipsychotics on glutamate levels in the ACC (25) and no relationship between baseline glutamatergic metabolites and treatment response (18).

We hypothesised that glutamate and Glx levels at baseline would be directly associated with treatment response following antipsychotic medication. However, we found that therapeutic effects as measured by the PANSS sub-scales were not associated with glutamate compounds at baseline. Previous ^1^H-MRS studies have shown glutamate metabolite levels vary depending on whether patients demonstrate a clinical response to antipsychotic treatment (15, 16, 21). However, these studies have been cross-sectional in design, which means it is not possible to determine whether a relationship with response suggests that glutamate levels are a predictor of response as opposed to a consequence of successful treatment. Our study was longitudinal in design and therefore addresses this limitation. The current study extends previous findings by reporting metabolites in ratio to CSF rather than Cr, a potential confounder in brain ^1^H-MRS studies carried out in schizophrenia patients (47).

We hypothesized glutamate and Glx levels would decrease after antipsychotic treatment relative to baseline. However, we found no effect of antipsychotics on glutamate and Glx levels in the ACC. A recent meta-analysis and systematic review (22) summarised 32 longitudinal studies investigating the effect of treatment on brain glutamate levels in schizophrenia. Four longitudinal studies have looked at glutamatergic changes in the ACC, three of which have reported no change in metabolites (25, 50, 51) and one study has reported a reduction in glutamate levels (24). Bustillo et al. (51) investigated the effect of antipsychotic medication in the ACC in minimally treated schizophrenia patients, with follow-up scans repeated after 1 (*n* = 10), 6 (*n* = 8), and 12 (*n* = 7) months. They reported no effect of time on glutamate, glutamine and Glx levels (CSF corrected) after antipsychotic medication (51). Similarly, Aoyama et al. investigated the effect of antipsychotics on glutamate and glutamine (CSF corrected) in the ACC of medication naive schizophrenia patients at baseline and repeated scans at 10 months (*n* = 14) and 80 months (*n* = 16). They reported at the 10-month follow-up one patient was on no medication, and at the 80-month scan, four of the patients were not taking any medication (50). Our study extends the findings from these studies by reporting results in a larger sample, as well as having parameters in place to assess concordance with antipsychotic medication. Kraguljac et al. investigated the effect of risperidone on Glx levels scaled to creatine in the ACC after 6 weeks of treatment (*n* = 61) and reported no reduction of Glx levels (25). Our study extends the findings from this study by scaling to water and correcting for CSF, as well as reporting results for both glutamate and Glx levels. Conversely, Egerton et al. reported a reduction in glutamate levels in the ACC after treatment with antipsychotic medication for 4 weeks (*n* = 46), however again this study scaled to Cr, whereas the current study reports both glutamate and Glx levels and scales to water and corrects for CSF (24). Overall, the results from the current study are in line with most of the observations carried out in the ACC and extend these by showing the lack of relationships is not due to confounding by alterations in creatine.

### Strengths and limitations

A strength of the study is the longitudinal design, and that metabolites were scaled to water and corrected for CSF content. We also use continuous scores for characterising symptom response to treatment, as opposed to dichotomising individuals in categories of responders and non-responders. The continuous symptom outcome has increased statistical power to detect a true relation with metabolite levels relative to a neat distinction between responders and non-responders, which could result in a loss of information.

A potential limitation is the heterogeneity in treatments administered to participants, as the differential effects of various antipsychotic medications on the glutamate system may have increased the variance in our data. However, all the antipsychotics were used at a dose that would block D_2/3_ receptors, which is thought to be the common mode of therapeutic action of these drugs (52). Additionally, the treatment reflects clinical practice, increasing the generalisability of our findings. Another potential limitation is that some patients had received antipsychotic treatment prior to the baseline scan. However, we excluded these subjects from the analysis of the effect of antipsychotic treatment on glutamatergic measures. Though our study has a relatively modest sample size, Bayesian statistical analyses provided moderate to anecdotal evidence in favour of the null findings. A further limitation is that using the PANSS for patients with bipolar disorder could have induced a floor effect, showing no change in negative symptoms after treatment. However, we chose the PANSS as it is a standardised scale for measuring psychopathology in a transdiagnostic sample of psychotic disorders. For example, PANSS indexes both positive and manic items. Furthermore, time to response has been subject to debate, with some studies suggesting non-response before 4 weeks is a predictor of subsequent non-response (40) and other suggesting treatment response at 4 weeks may be too early an interval in first-episode psychosis patients (53). However, it is unknown how generalizable the findings are from Gallego et al. as their participants were assigned to treatment with either olanzapine or risperidone (53). Whereas in our study the choice of antipsychotic commenced was determined by the treating clinician in discussion with the patient as per standard clinical practice. Another limitation of the current study is that there is no control group, although we would expect no changes in the PANSS scores of healthy volunteers, as they would not be treated with antipsychotic medication. However, a control group would be important to evaluate glutamatergic changes over time and this would be useful to investigate in a further study. Finally, macromolecules were not described in the basis set used for the ^1^H-MRS quantification and given that they represent a significant contamination source to the glutamate and Glx signal, future studies need to account for this to improve the accuracy of results (23, 54).

### Implications

Although glutamate has been implicated in the pathophysiology of schizophrenia, our findings indicate that the mechanism of action of antipsychotic medications does not have a marked effect on glutamatergic function in the ACC. Whilst we cannot exclude modest effects or an effect on other aspects of the glutamate system, this suggests that antipsychotics’ actions on other systems underlie their therapeutic effects (52). Moreover, the findings from the current study are not consistent with hypotheses that glutamate abnormalities underlie poor treatment response (55, 56) and further studies are needed to clarify this relationship.

## Conclusion

Using a longitudinal design, we report no effect of antipsychotics on ACC glutamate and Glx levels and no association between baseline ACC glutamate and Glx levels and clinical response in FEP patients. These data extend previous literature to indicate that antipsychotic efficacy is not primarily due to modulation of the glutamatergic system. Notably, other studies have used samples of exclusively non-affective psychosis patients, but ours includes a great proportion of bipolar disorder patients, which could cause discrepancies with prior findings. However, we think that using a transdiagnostic approach is more appropriate in the field of psychosis. For example, our group showed that dopamine dysregulation in psychotic disorders as well as determinants of treatment response cut across the traditional categories of affective and non-affective psychosis (32, 57). The present study may serve as an important reference for other studies which will likewise examine a sample encompassing affective and non-affective psychotic disorders. Finally, more studies are needed to clarify the relationship between antipsychotics, glutamate, and treatment response.

## Data availability statement

The raw data supporting the conclusions of this article will be made available by the authors, without undue reservation.

## Ethics statement

The studies involving human participants were reviewed and approved by the East of England-Cambridge East NHS Research Ethics Committee. The patients/participants provided their written informed consent to participate in this study.

## Author contributions

OH contributed to conception and design of the study. RAM, FB, SJ, FP, MN, MR, and PH contributed to organisation of the study, recruitment, and data collection. UZ performed the statistical analysis and wrote the first draft of the manuscript. MO and GM contributed to the statistical analysis. All authors contributed to manuscript revision, read, and approved the submitted version.

## Footnotes

1
http://s-provencher.com/lcm-manual.shtml
