# Corrigendum: The effect of antipsychotics on glutamate levels in the anterior cingulate cortex and clinical response: a ^1^H-MRS study in first-episode psychosis patients

**DOI:** 10.3389/fpsyt.2024.1292075

**Published:** 2024-03-01

**Authors:** Uzma Zahid, Robert A. McCutcheon, Faith Borgan, Sameer Jauhar, Fiona Pepper, Matthew M. Nour, Maria Rogdaki, Martin Osugo, Graham K. Murray, Pamela Hathway, Robin M. Murray, Alice Egerton, Oliver D. Howes

**Affiliations:** ^1^ Department of Psychosis Studies, Institute of Psychiatry, Psychology and Neuroscience, King’s College London, London, United Kingdom; ^2^ Department of Neuroimaging, Institute of Psychiatry, Psychology and Neuroscience, King’s College London, London, United Kingdom; ^3^ Department of Clinical and Movement Neurosciences, Queen Square Institute of Neurology, University College London Centre, London, United Kingdom; ^4^ Max Planck University College London Centre for Computational Psychiatry and Ageing Research, London, United Kingdom; ^5^ Wellcome Trust Centre for Neuroimaging, University College London, London, United Kingdom; ^6^ Department of Child and Adolescent Psychiatry, Institute of Psychiatry, Psychology and Neuroscience, King’s College London, London, United Kingdom; ^7^ Department of Psychiatry, University of Cambridge, Cambridge, United Kingdom; ^8^ Institute of Clinical Sciences, Faculty of Medicine, Imperial College London, London, United Kingdom; ^9^ H. Lundbeck UK, Valby, Denmark

**Keywords:** spectroscopy, NMDA, imaging and schizophrenia, CSF-correction, longitudinal, glutamate

In the published article, there was an error in the author list. Alice Egerton was erroneously excluded. The corrected author list appears above.

The authors apologize for this error and state that this does not change the scientific conclusions of the article in any way. The original article has been updated.

