# Supplementary material for: The effect of antipsychotics on glutamate levels in the anterior cingulate cortex and clinical response: A 1H-MRS study in first-episode psychosis patients
Source: Front Psychiatry. 2022 Aug 11;13:967941. doi: 10.3389/fpsyt.2022.967941 (PMC9403834; doi:10.3389/fpsyt.2022.967941)
Supplement: Supplementary file 1 [file Data_Sheet_1.docx]

**Supplementary Material**

Methods

Structural MRI Imaging

Images were acquired on a 3.0 Tesla Signa (GE) system at the Centre for Neuroimaging Sciences, IoPPN, London. 196 high-resolution T1-weighted images were acquired using a three-dimensional enhanced fast gradient echo sequence using the following scan parameters: repetition time – 6.98 msec, echo time – 2.85 msec, flip angle - 11°, matrix – 256 x 256, FoV – 260, slice thickness - 1.2 mm.

Results

S1. Glutamate and Glx levels (corrected for CSF) before and after antipsychotic administration in antipsychotic free individuals

There was no significant change between baseline (M = 12.76, SD = 2.35, n = 17) and follow-up glutamate levels ([M = 13.65, SD = 1.70, n = 17]; t(16)= -1.415, *p* = 0.176) or between baseline (M = 19.26, SD = 3.28, n = 17) and follow-up Glx levels ([M = 19.78, SD = 2.37, n = 17]; t(16)= -0.724, *p* = 0.479).

S2. Glutamine and NAA levels (corrected for CSF) before and after antipsychotic administration

There was no significant change between baseline (M = 6.41, SD = 2.56) and follow-up Glutamine levels ([M = 5.80, SD = 2.18]; t(21)= 1.051, *p* = 0.305) or between baseline (M = 11.44, SD = 1.43) and follow-up NAA levels ([M = 11.99, SD = 1.51]; t(21)= -1.91, *p* = 0.069).

S3. Association between change in the PANSS subscales and the change in glutamate and Glx levels (corrected for CSF)

There was no significant association between the change in glutamate levels from baseline to follow-up and the change from baseline to follow-up in PANSS positive scores (r=0.050, n=21, *p*=0.829), the follow-up PANSS negative scores (r=-0.269, n=21, *p*=0.239) the follow-up PANSS general scores (r=0.83, n=21, *p*=0.722) or the follow-up PANSS total scores (r=0.78, n=21, *p*=0.737). There was no significant association between Glx levels at follow-up and the follow-up PANSS positive scores (r=-0.298, n=21, *p*=0.190) the follow-up PANSS negative scores (r=-0.086, n=21, *p*=0.710) the follow-up PANSS general scores (r=-0.346, n=21, *p*=0.125) or the follow-up PANSS total scores (r=-0.293, n=21, *p*=0.198).

S4. Association between follow-up glutamate and Glx levels (corrected for CSF) and follow-up PANSS subscale scores

There was no significant association between glutamate levels at follow-up and the follow-up PANSS positive scores (r=-0.042, n=21, p=0.856), the follow-up PANSS negative scores (r=-0.271, n=21, p=0.235) the follow-up PANSS general scores (r=0.222, n=21, p=0.334) or the follow-up PANSS total scores (r=0.038, n=21, p=0.871). There was no significant association between Glx levels at follow-up and the follow-up PANSS positive scores (r=-0.251, n=21, p=0.273) the follow-up PANSS negative scores (r=-0.306, n=21, p=0.177) the follow-up PANSS general scores (r=-0.064, n=21, p=0.782) or the follow-up PANSS total scores (r=-0.202, n=21, p=0.380).

S5. Association between baseline glutamine and NAA levels (corrected for CSF) and the change in PANSS sub-scales scores

There was no significant association between glutamine levels at baseline and the change in PANSS positive scores (r = -0.259, n = 24, *p* = 0.223), the change in PANSS negative scores (r = -0.061, n = 24, *p* = 0.779) the change in PANSS general scores (r = -0.383, n = 24, *p* = 0.065) or the change in PANSS total scores (r = -0.277, n = 24, *p* = 0.191). There was no significant association between NAA levels at baseline and the change in PANSS positive scores (r = -0.103 n = 24, *p* = 0.631), the change in PANSS negative scores (r = -0.199, n = 24, *p* = 0.353), the change in PANSS general scores (r = -0.294, n = 24, *p* = 0.164) or the change in PANSS total scores (r = -0.236, n = 24, *p* = 0.266).

S6. Association between baseline creatine levels and the change in PANSS sub-scales scores

There was no significant association between creatine levels at baseline and the change in PANSS positive scores (r = -0.261, n = 24, *p* = 0.217), the change in PANSS negative scores (r = -141, n = 24, *p* = 0.510) the change in PANSS general scores (r = -0.334, n = 24, *p* = 0.110) or the change in PANSS total scores (r = -0.221, n = 24, *p* = 0.300).

| **Baseline** |  |  |  |  |  |  |
| --- | --- | --- | --- | --- | --- | --- |
| **Subject ID** | **CSF** | **GM** | **WM** | **Glu %SD** | **FWHM** | **S/N** |
| 5 | 0.229 | 0.678 | 0.093 | 6 | 0.038 | 21 |
| 6 | 0.000 | 0.862 | 0.138 | 6 | 0.038 | 22 |
| 8 | 0.207 | 0.608 | 0.185 | 9 | 0.048 | 10 |
| 10 | 0.197 | 0.719 | 0.084 | 6 | 0.038 | 23 |
| 12 | 0.238 | 0.676 | 0.086 | 9 | 0.033 | 12 |
| 14 | 0.257 | 0.652 | 0.091 | 6 | 0.038 | 23 |
| 15 | 0.157 | 0.683 | 0.160 | 9 | 0.033 | 21 |
| 17 | 0.227 | 0.686 | 0.087 | 6 | 0.033 | 25 |
| 21 | 0.248 | 0.603 | 0.149 | 6 | 0.038 | 19 |
| 24 | 0.242 | 0.694 | 0.064 | 5 | 0.057 | 18 |
| 25 | 0.193 | 0.655 | 0.152 | 5 | 0.029 | 29 |
| 27 | 0.220 | 0.695 | 0.084 | 6 | 0.033 | 25 |
| 30 | 0.276 | 0.624 | 0.099 | 8 | 0.033 | 13 |
| 33 | 0.265 | 0.626 | 0.109 | 7 | 0.043 | 17 |
| 34 | 0.245 | 0.625 | 0.131 | 8 | 0.043 | 17 |
| 37 | 0.273 | 0.645 | 0.082 | 6 | 0.033 | 20 |
| 48 | 0.178 | 0.687 | 0.136 | 6 | 0.043 | 16 |
| 61 | 0.229 | 0.680 | 0.091 | 11 | 0.053 | 8 |
| 65 | 0.327 | 0.616 | 0.057 | 7 | 0.043 | 16 |
| 71 | 0.196 | 0.731 | 0.073 | 7 | 0.048 | 19 |
| 74 | 0.229 | 0.697 | 0.074 | 7 | 0.043 | 18 |
| 75 | 0.292 | 0.590 | 0.118 | 7 | 0.033 | 20 |
| 78 | 0.146 | 0.734 | 0.120 | 5 | 0.033 | 28 |
| 82 | 0.258 | 0.618 | 0.124 | 8 | 0.033 | 21 |
| 83 | 0.217 | 0.689 | 0.094 | 13 | 0.057 | 15 |
| 2225 | 0.206 | 0.666 | 0.128 | 6 | 0.038 | 20 |
|  |  |  |  |  |  |  |
| **Follow-up** |  |  |  |  |  |  |
| **Subject ID** | **CSF** | **GM** | **WM** | **Glu %SD** | **FWHM** | **S/N** |
| 5 | 0.156 | 0.693 | 0.150 | 7 | 0.038 | 15 |
| 6 | 0.090 | 0.758 | 0.152 | 7 | 0.029 | 27 |
| 8 | 0.240 | 0.659 | 0.102 | 8 | 0.038 | 14 |
| 12 | 0.206 | 0.704 | 0.090 | 6 | 0.038 | 23 |
| 14 | 0.299 | 0.616 | 0.085 | 7 | 0.043 | 17 |
| 15 | 0.182 | 0.677 | 0.141 | 6 | 0.033 | 24 |
| 17 | 0.211 | 0.681 | 0.108 | 7 | 0.038 | 25 |
| 21 | 0.256 | 0.611 | 0.133 | 7 | 0.038 | 20 |
| 25 | 0.248 | 0.650 | 0.102 | 6 | 0.033 | 25 |
| 27 | 0.215 | 0.704 | 0.082 | 7 | 0.029 | 19 |
| 30 | 0.286 | 0.601 | 0.113 | 9 | 0.038 | 12 |
| 31 | 0.205 | 0.606 | 0.189 | 7 | 0.048 | 14 |
| 34 | 0.253 | 0.613 | 0.133 | 8 | 0.033 | 17 |
| 37 | 0.306 | 0.622 | 0.071 | 6 | 0.033 | 20 |
| 48 | 0.187 | 0.675 | 0.138 | 7 | 0.053 | 15 |
| 61 | 0.227 | 0.684 | 0.089 | 8 | 0.033 | 15 |
| 65 | 0.305 | 0.623 | 0.072 | 10 | 0.048 | 14 |
| 71 | 0.244 | 0.661 | 0.095 | 8 | 0.033 | 20 |
| 74 | 0.218 | 0.673 | 0.109 | 7 | 0.024 | 22 |
| 78 | 0.224 | 0.659 | 0.117 | 5 | 0.029 | 27 |
| 83 | 0.253 | 0.661 | 0.085 | 7 | 0.043 | 20 |
| 2225 | 0.192 | 0.732 | 0.075 | 6 | 0.038 | 24 |

Supplementary Table 1 The individual tissue fractions (white matter [WM], gray matter [GM], cerebrospinal fluid [CSF]), full-width at half-maximum (FWHM), Cramér-Rao lower bounds (CRLB) for glutamate, and signal to noise (S/N) ratio at baseline and follow up.


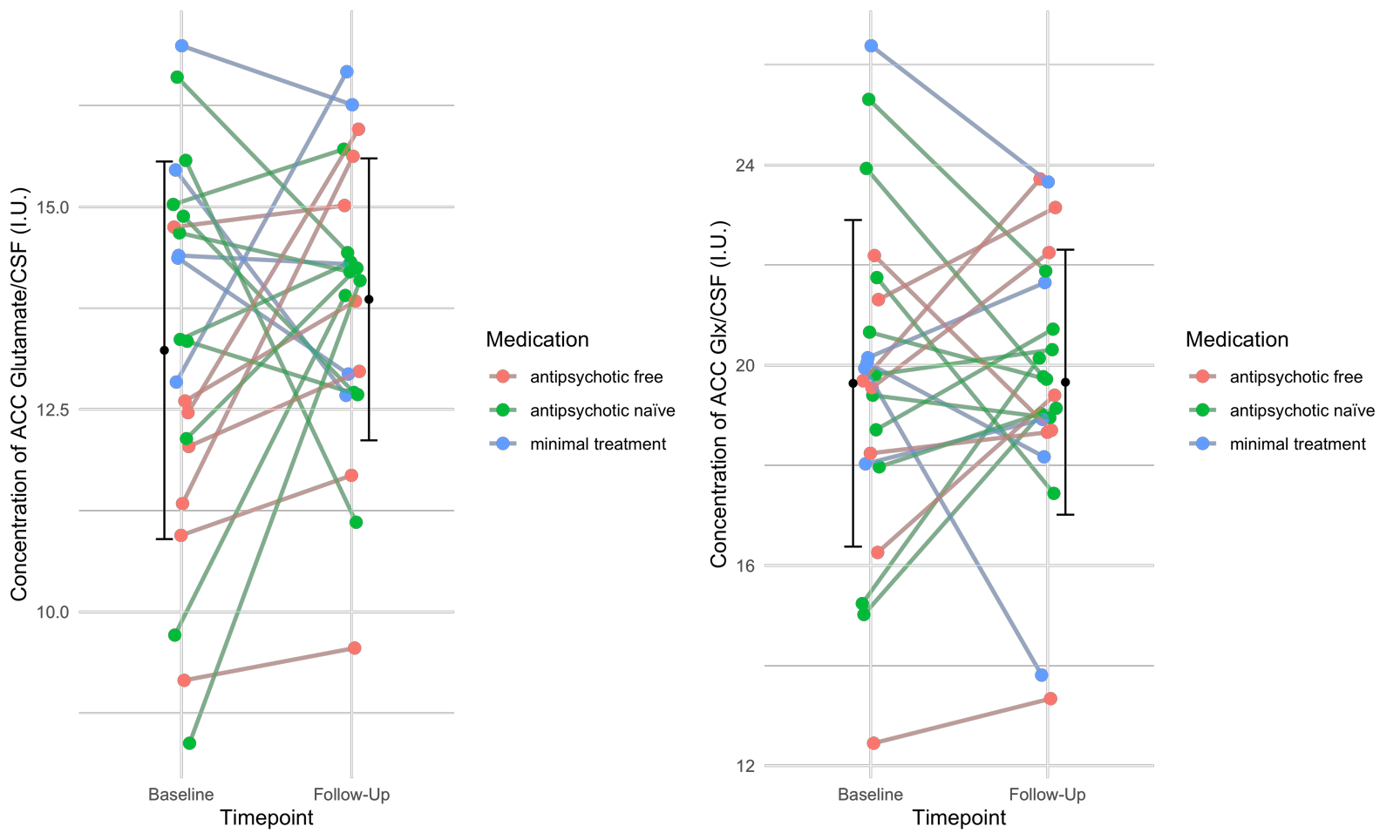


Figure 1 Left: The individual change in glutamate levels from baseline to follow-up, with group mean (SD) of glutamate levels at baseline and follow-up (black circle and error bars). Individual change in the figure is stratified by medication status. Results of the paired t-test indicated no significant difference in glutamate levels over time (p = 0.260). Right: The individual change in Glx levels from baseline to follow-up, with the group mean (SD) Glx levels at baseline and follow-up (black circles and error bars). Individual change in the figure is stratified by diagnosis. Results of the paired t-test indicated no significant difference in Glx levels over time (p = 0.973).


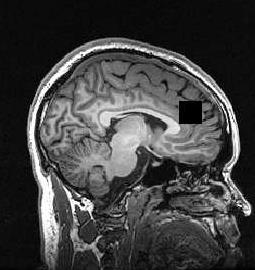


Supplementary figure 2 Depicting the placement of the voxel over the anterior cingulate during the ^1^H-MRS scan.


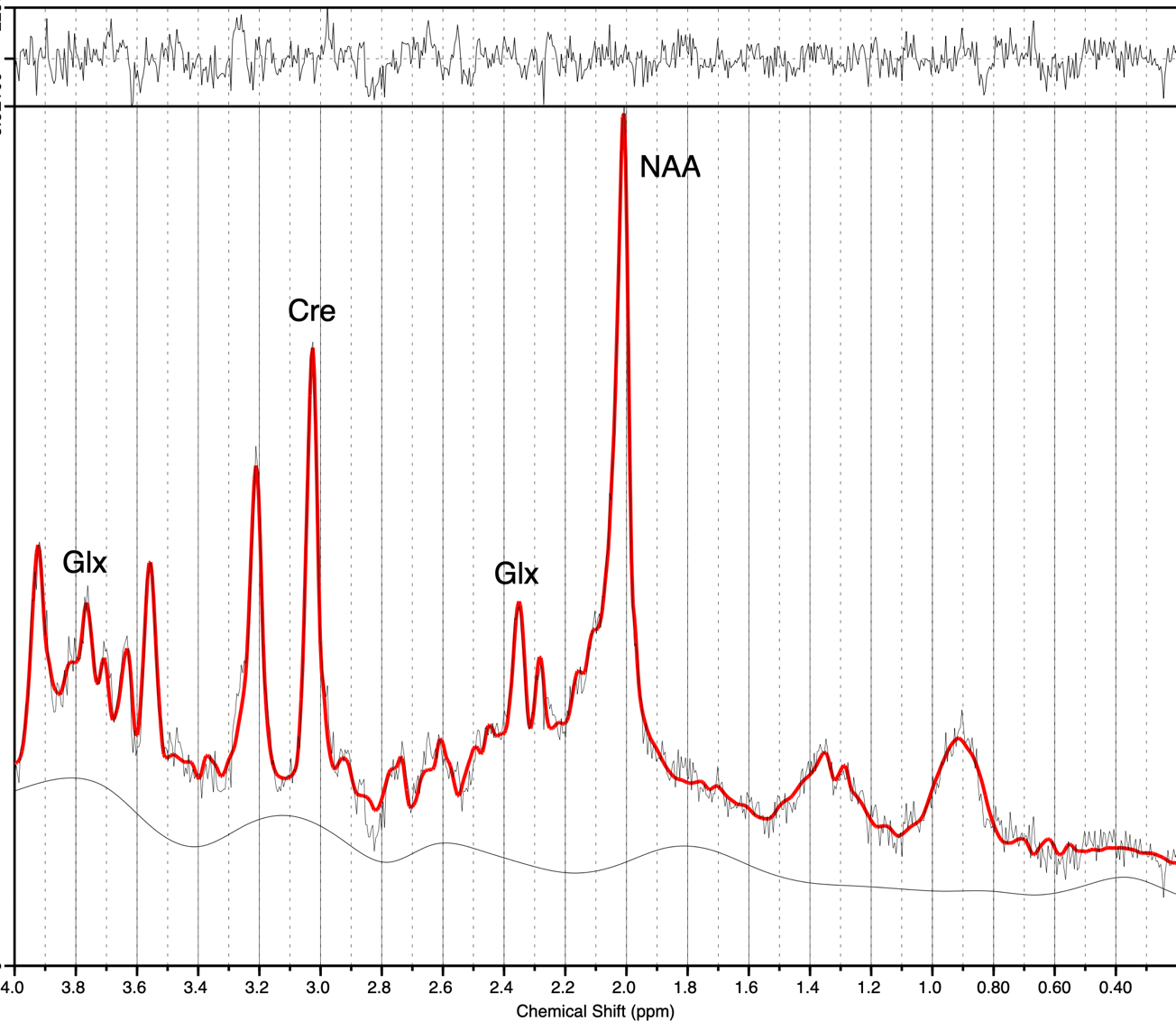


Supplementary figure 3 Shows a sample spectrum acquired from ^1^H-MRS quantification. Selected metabolite peaks are indicated. Glx = combined signal of glutamate and glutamine; Cre = Creatine; and NAA = N-acetylaspartate.
